# Response to HAART according to sex and origin (immigrant vs autochthonous) in a cohort of patients who initiate antiretroviral treatment

**DOI:** 10.1186/1758-2652-13-S4-P21

**Published:** 2010-11-08

**Authors:** JA Perez-Molina, M Mora-Rillo, I Suarez-Lozano, JL Casado, R Teira, P Rivas, E Pedrol, A Hernando, P Domingo, E Barquilla, H Esteban, J Gonzalez-Garcia

**Affiliations:** 1 Hospital Ramón y Cajal, Tropical Medicine Unit. Infectious Diseases Dpt., Madrid, Spain; 2 Hospital La Paz, Madrid, Spain; 3 Cohorte VACH, Huelva, Spain; 4 Hospital Ramon y Cajal, Madrid, Spain; 5 Cohorte VACH, Torrelavega, Spain; 6 Hospital Carlos III, Madrid, Spain; 7 Cohorte VACH, Tarragona, Spain; 8 Hospital 12 de Octubre, Madrid, Spain; 9 Cohorte VACH, Barcelona, Spain; 10 Fundación SEIMC-GESIDA, Madrid, Spain

## Purpose

Although poorly studied, gender differences can affect the efficacy of HAART. Immigrant women (IW) may also be at risk of treatment failure due to greater marginalization, cultural differences, or reduced access to health care. This subanalysis examined differences in baseline characteristics and response to HAART according to sex and geographic origin.

## Methods

Subanalysis of GES-5808 (retrospective comparative study autochthonous/immigrant patients initiating HAART Jan05-Dec06). Late diagnosis was defined as a CD4+ count ≥200, and/or AIDS at initiation of HAART. The primary endpoint was time to treatment failure (TTF), which was defined as virological failure (VF), death, opportunistic infection (OI), interruption of HAART, or loss to follow-up. Survival was analyzed using a univariate (Kaplan-Meier) and multivariate (Cox regression) approach.

## Results

Patient Characteristics at Initiation of HAART (Table 1)

**Table 1:** 

	WOMEN (318)	MEN (772)	P
Immigrants, %	45.6	31.1	<0.001

Age, years (IQR)	35 (29-41)	39 (33-44)	<0.001

Median viral load (IQR)/CD4 (IQR)	4.7 (4.2-5.2)/ 217 (113-300)	5.0 (4.5-5.4)/ 190 (69-280)	0.001/0.002

Coinfection with HBV or HCV %	25.2	29.3	0.32

Stage C/Late diagnosis, %	21.2/49.0	29.0/59.0	0.006/0.003

Median time from diagnosis of HIV infection to initiation of HAART, mo (IQR)	15 (2-43)	16 (2-49)	0.55

Median TTF, wk	147	171	<0.001

VF/OI, %	5.3	6.3	0.52

Educational level and occupational status were significantly poorer in women. The adjusted risk of treatment failure in women was not significantly different from that of men (HR, 1.101; 95% CI, 0.79-1.53). The increase in CD4 lymphocytes was equivalent (185 vs 205). TTF was shorter among (IW) than autochthonous women (AW): 124 weeks (95% CI, 64-183) vs 152 (95% CI, 127-174). Most immigrant women were African and Latin American, and their dropout rate (25.5 vs 11.6) was double that of AW.

## Conclusions

Response to HAART was similar in both sexes. Men started HAART later and women had higher loss to follow-up and more treatment switches. This was even more common among IW. Earlier diagnosis is necessary for men; measures to improve adherence should be promoted among women, especially IW.

